# Ambient Air Pollution and Depressive Symptoms in Older Adults

**DOI:** 10.1289/ehp.1409657

**Published:** 2015-05-01

**Authors:** Yongqing Gao, Tan Xu, Wenjie Sun

**Affiliations:** 1 School of Food Science, Guangdong Pharmaceutical University, Zhongshan, Guangdong, China; 2 School of Public Health and Jiangsu Key Laboratory of Preventive and Translational Medicine for Geriatric Diseases, Medical College of Soochow University, Suzhou, Jiangsu, China; 3 School of Public Health and Tropical Medicine, Tulane University, New Orleans, Louisiana, USA

Wang et al. (2014) investigated the relationship between ambient air pollution and depressive symptoms in a prospective study of elderly people over age 65 years. We are very surprised that the authors found no association between ambient air pollution and depressive symptoms, which is inconsistent with most previous studies (e.g., Banerjee et al. 2012; Calderón-Garcidueñas et al. 2014). A study of a population of elderly men indicated one potential biological mechanism may be methylation, which was decreased after acute exposure to fine particulate matter (Madrigano et al. 2012).

We identified three issues with the study by Wang et al. (2014). First, these authors used outdoor air pollution as the exposure of interest. Long-term exposure was estimated based on residential distance from the nearest major road. Considering only residential distance to the nearest major roadway, without taking into account other sources of exposure, may be insufficient to accurately estimate long-term exposure to traffic pollution. Short-term exposure to ambient air pollution was estimated based on pollutant levels measured at only one monitoring site, the Harvard–U.S. Environmental Protection Agency Supersite stationary ambient monitoring site. Although the monitoring site was located < 20 km from the home of any study participant, the number of monitoring sites was insufficient. Thus, participants’ exposures to ambient pollutant might be misclassified. Moreover, considering that the participants were elderly, they were less likely than other groups to spend time outdoors (Kerr et al. 2012). Hence, outdoor air pollution might not reflect their true exposures. Lacking validation of these estimated exposures, the negative conclusion is unconvincing.

Second, the Revised Center for Epidemiologic Studies Depression Scale (Eaton et al. 2004) is not the first choice for measuring depressive symptoms in the elderly; the Geriatric Depression Scale (Yesavage et al. 1982) is more suitable. The evidence from clinical diagnosis of depression shows a positive association between air pollution and depression. For example, Cho et al. (2014) found that ambient air pollution was positively associated with emergency department visits for depressive episodes among 4,985 Koreans using clinical diagnosis of depression episode as outcome. Of note, clinical diagnosis of depression is a more objective measure than depressive symptoms.

Third, Wang et al. did not address health status in their reported association. There is convincing evidence supporting the association between cardiovascular disease (CVD) and both ambient air pollution (e.g., Grahame and Schlesinger 2010) and depression (e.g., Sun et al. 2013). Hence, previous history of CVD could confound or modify the association between ambient air pollution and depressive symptoms. Stratified analysis according to health or CVD status is warranted. Without such analysis, the negative conclusion is not well supported.

## References

[r1] Banerjee M Siddique S Dutta A Mukherjee B Ranjan Ray M 2012 Cooking with biomass increases the risk of depression in pre-menopausal women in India. Soc Sci Med 75 3 565 572; 10.1016/j.socscimed.2012.03.021 22580071

[r2] Calderón-Garcidueñas L Calderón-Garcidueñas A Torres-Jardón R Avila-Ramirez J Kulesza RJ Angiulli AD 2014 Air pollution and your brain: what do you need to know right now. Prim Health Care Res Dev 1 17; 10.1017/S146342361400036X 25256239

[r3] Cho J Choi YJ Suh M Sohn J Kim H Cho SK 2014 Air pollution as a risk factor for depressive episode in patients with cardiovascular disease, diabetes mellitus, or asthma. J Affect Disord 157 45 51; 10.1016/j.jad.2014.01.002 24581827

[r4] Eaton WW, Smith C, Ybarra M, Muntaner C, Tien
A. (2004). Center for Epidemiologic Studies
Depression Scale: review and revision (CESD and CESD-R). In: The Use of
Psychological Testing for Treatment Planning and Outcomes Assessment, Part
3rd (Maruish ME, ed).

[r5] Grahame TJ Schlesinger RB 2010 Cardiovascular health and particulate vehicular emissions: a critical evaluation of the evidence. Air Qual Atmos Health 3 1 3 27; 10.1007/s11869-009-0047-x 20376169 PMC2844969

[r6] Kerr J, Sallis JF, Saelens BE, Cain KL, Conway TL, Frank LD, et al. 2012 Outdoor physical activity and self rated health in older adults living in two regions of the U.S. Int J Behav Nutr Phys Act 9 89; 10.1186/1479-5868-9-89 22846594 PMC3464785

[r7] Madrigano J Baccarelli A Mittleman MA Sparrow D Spiro A III Vokonas PS 2012 Air pollution and DNA methylation: interaction by psychological factors in the VA Normative Aging Study. Am J Epidemiol 176 3 224 232; 10.1093/aje/kwr523 22798479 PMC3491965

[r8] Sun WJ Xu L Chan WM Lam TH Schooling CM 2013 Are depressive symptoms associated with cardiovascular mortality among older Chinese? A cohort study of 64,000 people in Hong Kong. Am J Geriatr Psychiatry 21 11 1107 1115; 10.1016/j.jagp.2013.01.048 23567371

[r9] Wang Y Eliot MN Koutrakis P Gryparis A Schwartz JD Coull BA 2014 Ambient air pollution and depressive symptoms in older adults: results from the MOBILIZE Boston study. Environ Health Perspect 122 6 553 558; 10.1289/ehp.1205909 24610154 PMC4050499

[r10] Yesavage JA Brink TL Rose TL Lum O Huang V Adey M 1982 Development and validation of a geriatric depression screening scale: a preliminary report. J Psychiatr Res 17 1 37 49; PMID:7183759 10.1016/0022-3956(82)90033-4

